# Theoretical Analysis of the Radiation-Induced Conductivity in Polymers Exposed to Pulsed and Continuous Electron Beams

**DOI:** 10.3390/polym12030628

**Published:** 2020-03-09

**Authors:** Andrey P. Tyutnev, Vladimir S. Saenko, Aleksey D. Zhadov, Dmitriy A. Abrameshin

**Affiliations:** National Research University Higher School of Economics, 20 Miasnitskaya Ulitsa, Moscow 101000, Russia; atyutnev@hse.ru (A.P.T.); azhadov@hse.ru (A.D.Z.); dabrameshin@hse.ru (D.A.A.)

**Keywords:** radiation-induced conductivity, polymers, numerical calculations, multiple trapping model, time-resolved transient currents, transit time effects

## Abstract

We have performed comparative numerical calculations using a multiple trapping (MT) formalism with an exponential and an aggregate two-exponential trap distributions for describing two mostly used experimental setups for studying the radiation-induced conductivity (RIC) and the time-of-flight (TOF) effects. Computations have been done for pulsed and long-time electron-beam irradiations in a small-signal regime. Predictions of these two approaches differ appreciably in both setups. The classical MT approach proved very popular in photoconductive polymers generally and in molecularly doped polymers in particular, while a newly proposed complex MT worked well in common polymers. It has been shown that the complex MT successfully accounts for the presence of inherent deep traps, which may or may not have an energy distribution.

## 1. Introduction

Charge carrier transport in disordered organics has been extensively studied for almost 50 years starting from 1970 [1]. Early investigations in this field were confined to photoconductive materials using the time-of-flight (TOF) technique with pulsed photo excitation. It was discovered that the charge carrier transport in polyvinylcarbazole (PVK) and molecularly doped polymers (MDPs), widely used in electrophotographic industry, was *dispersive* so that the post-flight TOF current decay was an algebraic (slow) rather than an exponential (fast) observed in crystalline solids [2]. As a result, a mobility (called the drift mobility) should have been defined through an operational procedure by an intersection of the pre- and post-flight branches of a TOF curve presented in lg *j* – lg *t* coordinates [3]. Continuous-time random walk theory of Scher and Montroll [3] (a theory of choice in late 70s and early 80s) coexisted with a quasi-band multiple trapping (MT) formalism [4,5] but later both were superseded by the Gaussian disorder model (GDM) of the hopping transport developed by Bässler [6]. All these results have been outlined in a book [7]. The dipolar glass model (DGM) suggested in 1998 [8] is surely to be noted.

Currently, the main interest of researchers shifted to a group of disordered organics employed in such electronic devices as light emitting diodes, photovoltaic cells, field-effect transistors and memory devices. Transport of charge carriers, mostly injected from electrodes, was now studied by using the finished parts of these devices trying to reproduce their operational characteristics by selecting model parameters. Besides, organic films were very thin (up to several nanometers) and the notion of the drift mobility as an averaged quantity became unproductive.

Common polymers are mainly used as dielectrics in electrical and electronic devices. For engineers, charge carrier mobility in polymers was not a main point of interest compared with their breakdown strength and durability under high electric stress. The situation changed with the advent of nuclear weapons/power stations and spacecraft technology. Needs of all these industries required developing a transport theory in common polymers because a simplistic accumulation of empirical data on the radiation-induced conductivity (RIC) systemized on the empirical level could not naturally satisfy community demand. Model development was greatly alleviated by the fact that time of flight (TOF) effects were usually minor compared with the recombination role.

In its contemporary form, the RIC theory employs the quasi-band multiple trapping formalism using an exponential trap distribution and became known as the Rose-Fowler-Vaisberg (RFV) model [9]. Numerical calculations can also accommodate the Gaussian trap distribution with whatever values of energetic parameters for both trap distributions [10].

An important turning point in these studies occurred during 2006–2017 when Tyutnev et al. (see our latest paper [11]) developed a novel TOF technique; the so-called radiation induced variant employing pulses of 3 to 50 keV electrons provided by an electron gun ELA-65 (Orion, Moscow, Russia). In fact, this technique is a combination of three main modifications: TOF variant using 3 to 7 keV electrons producing carrier generation near an irradiated sample surface like in photo excitation; TOF-2 variant employing 50 keV electrons (the maximum energy available at the moment) for carrier generation in the bulk and TOF-1a method with the varying generation layer thickness. The main result of these investigations was that the carrier transport in MDPs was indeed moderately dispersive despite the fact that TOF curves in MDPs featured a horizontal plateau which in turn has been shown to be an artifact of a TOF technique (both photo- and radiation-induced) due to surface layers with a depleted dopant concentration.

For interpretation of these results, we used a conventional MT formalism with an exponential or the Gaussian trap distributions supplemented with appropriate computer codes. Eligibility of these MT models in describing hopping carrier transport in terms of the quasi-band theory seems to be solved positively based on a transport level concept which is currently under intensive development [12].

But our recent investigations of the radiation-induced conductivity in commercial polymers (a legitimate representative of disordered organics) using pulsed and continuous step-function irradiations have shown that the above picture of the charge carrier transport described by the one-parameter exponential or the Gaussian distributions of hopping centers (respectively, traps) needs reexamination [13]. Indeed, PVK and MDPs generally follow predictions of the conventional MT theories concerning both RIC and TOF experiments. The situation changes for films of polyethyleneterephthalate (PET), polyimide (PI), polystyrene (PS) and others. To describe their RIC, one needs using an aggregate trap distribution combining two exponentials as first suggested in the cited work.

In the present paper, we plan to carry out numerical calculations of both the RFV and the modified RFV (RFVm) models in a broad range of irradiation times and electric fields and to apply these results for interpretation of the published experimental data addressing some ambiguities.

## 2. Models Formulation

The RFV and RFVm models both employ the MT formalism whose basic equations are as follows:

(1) $$\partial \rho /\partial t=\left(N_{0}/\tau_{0}\right)\left[M\left(E\right)-\rho \right]/M_{0}-\rho \nu_{0}\exp\left(-\frac{E}{kT}\right)$$

(2) $$N=N_{0}+\int_{0}^{\infty }\rho dE$$

Here, *N* is the total concentration of the mobile carriers (in our case, electrons). *N*_0_ is their concentration in conduction zone where they have the microscopic mobility *μ*_0_ and lifetime *τ*_0_. Energy trap distribution is *M*(*E*), total trap concentration is *M*_0_ (note that trap energy is taken to be positive). Distribution function of trapped carriers is given by *ρ*(*E*). Also, *ν*_0_ is the frequency factor, T-temperature, k-the Boltzmann constant. Here, variables *N*_0_, *N* and *ρ* are functions of time only and refer naturally to the RIC experiment. In a TOF setup, these variables become *x*-coordinate dependent (1D geometry is assumed).

Now, we have to specify the carrier generation/loss terms and formulate appropriate continuity equations. Below, the generation rate of quasi-free electrons is *g*_0_ which is assumed to be constant and uniform during irradiation time *t_g_* and is zero afterwards. A continuity equation in RIC may be written like this:

(3) $$dN/dt=g_{0}-k_{rec}N_{0}N.$$

In a TOF setup when carrier exit to electrodes overrides recombination losses, it should be changed:

(4) $$\partial N(x,t)/\partial t+\mu_{0}F_{0}\partial N_{0}(x,t)/\partial x=g_{0},$$

implying that now the concentration becomes necessarily coordinate-x dependent (*F*_0_ is an applied field which is still uniform and constant). The generation rate has the previous meaning. As a consequence, TOF method becomes TOF-2 method.

Initial conditions for both setups are the same: at *t* = 0 all concentrations are equal to zero. Boundary conditions for the TOF-2 case are specific and may be found in literature [4,14]. The output quantities of these calculations are the radiation-induced conductivity which is equal to

(5) $$\gamma_{r}\left(t\right)=e\mu_{0}N_{0}\left(t\right),$$

and the current density in a TOF-2 case (the concept of conductivity is not applicable here)

(6) $$j\left(t\right)=\frac{e\mu_{0}F_{0}}{L}\int_{0}^{L}N_{0}\left(x,t\right)dx,$$

where *e*-an elementary electric charge and *L*-a film thickness.

At last, we have to specify a trap energy distribution. The conventional RFV model uses a simple exponential

(7) $$M\left(E\right)=\frac{M_{0}}{E_{1}}\exp\left(-E/E_{1}\right),$$

where *E*_1_ is in fact an average trap energy. Dispersion parameter α = kT/*E*_1_ controls RIC current shapes for step-function irradiation in a small-signal regime [4,9,14]. As mentioned earlier, for α ≤ 0.5 there are even closed-form expressions for these current shapes [14]. In the RFVm model, the above trap distribution extends only to a separation energy *E*_s_. For *E* ≥ *E*_s_ the distribution parameter of the second exponential *E*_2_ rises appreciably. Now, we have to deal with two dispersion parameters: α_1_ (the former α) and α_2_ = kT/*E*_2_. The idea of this separation is that each of two trap fractions clearly identifies its contribution to the RIC as suggested in References [10,13]. An explicit form of *M*(*E*) is as follows

$$M\left(E\right)=\frac{{\hat{M}}_{0}}{E_{1}}\exp\left(-E/E_{1}\right),\;E<E_{s}$$

(8) $$M\left(E\right)=\frac{{\hat{M}}_{0}}{E_{1}}\exp\left(-E_{s}/E_{1}\right)\exp\left[\left(E-E_{s}\right)/E_{2}\right],E\geq E_{s}$$

where ${\hat{M}}_{0}=M_{0}{\left[1+\left(\frac{E_{2}}{E_{1}}-1\right)\exp\left(-E_{s}/E_{1}\right)\right]}^{-1}$

For reference, we indicate that the relative fraction of deep traps is equal to

(9) $$\eta =M_{2}/M_{0}=\frac{\left(E_{2}/E_{1}\right)\exp\left(-E_{s}/E_{1}\right)}{1+\left(E_{2}/E_{1}-1\right)\exp\left(-E_{s}/E_{1}\right)}$$

and for $\exp\left(-E_{s}/E_{1}\right)\ll 1$ we have

(10) $$\eta \approx \frac{E_{2}}{E_{1}}\exp\left(-E_{s}/E_{1}\right).$$

Here, *M*_2_ is the concentration of deep traps with energies exceeding *E*_s_. It should be noted that in the framework of the RFVm model as in the RFV one, the RIC prompt component ${\overset{⌢}{\gamma }}_{p}=g_{0}\mu_{0}\tau_{0}e$ (*e* is an electronic charge) is field dependent duplicating field dependence of *g*_0_ contrary to the genuine RIC prompt conductivity which is field independent. So, model parameters should be found by fitting experimental and numerical current curves of the RIC *delayed components* (below, RICd curves). In thin samples stressed by high fields, this approach converts into a TOF-2 setup.

## 3. Model Parameters

As a prototype polymer for numerical simulations we have selected polyethyleneterephthalate (PET) as the best documented polymer regarding its RIC and pulsed small-signal irradiations (with a near step-function time profile) in particular. Also, we chose to employ the tentative model parameters suggested in Reference [13]. These are as follows: α = 0.5, *μ*_0_ = 10^−5^ m^2^/(V s), *τ*_0_ = 2 × 10^−11^ s, *ν*_0_ = 3 × 10^7^ s^−1^, *k*_rec_ = 5.8 × 10^−14^ m^3^·c^−1^ and *M*_0_ = 10^26^ m^−3^. Also, at *F*_0_ = 4 × 10^7^ V/m (the field employed in the cited work), we have *g*_0_ (m^−3^·s^−1^) = 6.24 × 10^19^ *R*_0_ (Gy/s) relating theoretical predictions and experimental conditions. By changing properly such parameters as *η*, *α*_2_ and *E*_s_ it is easy to mimic a specific polymer, but the general behavior predicted by the RFV and RFVm models does not depend on these details.

## 4. Computation Results

### 4.1. *τ*_c_ -Approximation

The most interesting results relate to PET commercial films irradiated in a small-signal regime in a broad range of irradiation times from 3 ns to 9 min [13,15,16]. In this polymer, the effect of deep traps may be represented by a single parameter *τ*_c_ accounting for a monomolecular trapping of mobile carriers (in PET, electrons [17]) allowing no thermal detrapping. This circumstance allows employing analytical formulas developed in Reference [14] and already effectively used in Reference [13]. It has been shown that *τ*_c_ = *τ*_0_ (*M*_0_/*M*_2_) [13] but this quantity is better considered as a fitting parameter.

**Figure 1:**
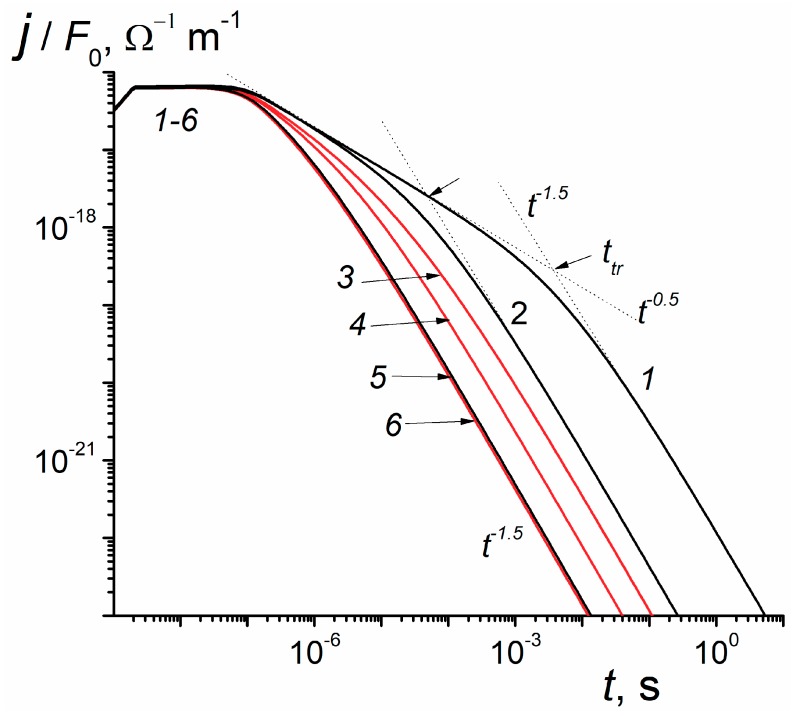
Computed time-of-flight (TOF)-2 curves for a prototype polymer reduced to a unit electric field. Pulse length 2 ns, film thickness 2.5 μm. *τ*_c_ = 5.4 × 10^3^ s (1,2,5, black) and *τ*_c_ = 5.4 × 10^−10^ s (3,4,6, red); electric field 10^7^ (1,3), 10^8^ (2,4) and 10^9^ V/m (5,6). For *t* ≤ 2 ns, results refer to radiation-induced conductivity (RICd) only. Also, *g*_0_ = 10^20^ m^3^·c^−1^ and *k*_rec_ is assumed zero. Note that curves (5) and (6) almost coincide.

Figure 1 presents results of TOF-2 calculations which have been done using the formulas and the MathCad packet given in Reference [13]. It is important to note that *τ*_c_-approximation allows assessing transit time effects in the presence of deep trapping. An essential requirement is the absence of recombination (for irradiation time 2 ns used in Figure 1, this condition is easily fulfilled).

We consider two groups of current transients, the first with *τ*_c_ = 5.4 × 10^3^ s effectively excluding deep traps and the second with the above value of *τ*_c_ = 5.4 × 10^−10^ s. Curves of the first group are the well-known TOF-2 transients relating to the conventional dispersive transport with a dispersion parameter α = 0.5 [9,14]. It is seen that transit times are easily determined for *F*_0_ = 10^7^ V/m (curve 1), not so easily for *F*_0_ = 10^8^ V/m (curve *2*) and cannot be determined at all for *F*_0_ = 10^9^ V/m (curve *5*). Transit times are 4.0 and 0.049 ms for curves (1) and (2) respectively while for curve (*5*) transit time could not be found as the pre-flight part of the TOF-2 curve simply disappears.

Curves of the second group defy transit time determination. At small fields TOF-2 curves (not shown in the Figure) tend to group around curve 3. High fields shift these curves to the left suggesting a kind of a TOF effect. Note that for all current transients asymptotic decay follows an algebraic law *t*^−1.5^ which is expected for a dispersive transport with α = 0.5 with or without deep traps present [9,14]. Such data should be given a careful consideration while processing numerical curves trying to extract information about drift mobility (see Discussion).

The near horizontal current plateau extending from the pulse end to about 30 ns in all current transients is nothing but an initial mobility plateau whose length *t*_pl_ is related to *ν*_0_ by the following formula *t*_pl_*ν*_0_ ≈ 1 suggested in paper [18]. It is interesting to note that the value of *ν*_0_ ≈ 3.3 × 10^7^ s^−1^ following from the above relationship is close to its value adopted in a set of model parameters presented earlier in Section 3. This relationship has been directly verified in the cited paper for the low density polyethylene (LDPE). In Reference [18], this procedure has been employed for directly determining the frequency factor in polymers featuring initial mobility plateau under irradiations with rectangular low intensity electron-pulses.

**Figure 2:**
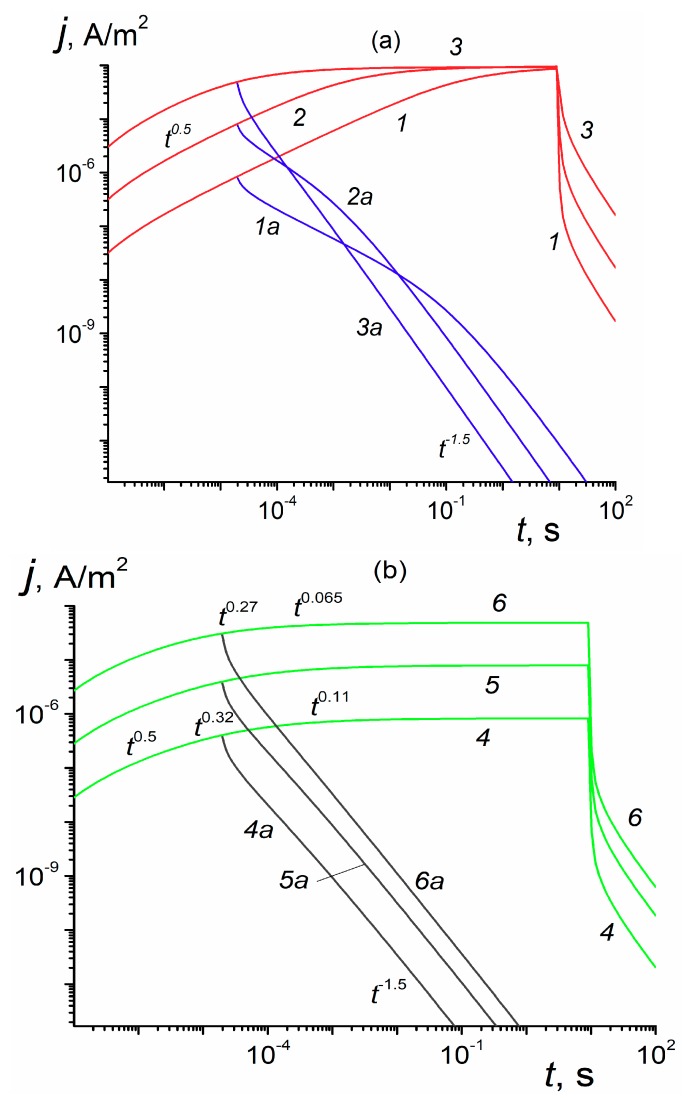
Calculated TOF-2 build-up and decay curves for a prototype polymer taken for two *τ*_c_: 5.4 × 10^3^ (**a**) and 5.4 × 10^−10^ s (**b**). Also, irradiation times 20 μs (1a–6a) and 10 s (1–6) and electric fields 10^7^ (1,1a,4,4a), 10^8^ (2,2a,5,5a) and 10^9^ V/m (3,3a,6,6a); *g*_0_ = 10^20^ m^−3^·s^−1^. Recombination neglected, film thickness 12 μm.

Now, we extend our consideration to a more prolonged irradiations 20 μs and 10 s long (Figure 2). Data for 20 μs pulses resemble those presented in Figure 1 while at a prolonged irradiation all curves approach a steady-state condition. We see that in case of the RFV model (Figure 2a) all curves approach the same value $j_{sat}=\left(\frac{1}{2}\right)g_{0}L_{}e$ ≈ 10${}^{-4}$ A/m^2^ accounting for a full charge collection by electrodes. The factor (1/2) accounts for a one-carrier (electron) conduction. The rate of approach increases with the field. According to the RFVm model (Figure 2b), the steady-state value *j*_st_ strongly depends on the field and should reach *j*_sat_ in the limit $F_{0}\to \infty$ (even at 10^9^ V/m *j*_st_ is only 4.8 × 10^−5^ A/m^2^ (≈ 0.48 *j*_sat_) as curve (6) in Figure 2b demonstrates).

At fields smaller than 10^7^ V/m, the stationary current density scales with the field. The logarithmic slope of the build-up curves *β* = dlg*j*/dlg*t* being around 0.5 at 1 μs slowly diminishes to zero at long times (as indicated in Figure 2b at appropriate times).

### 4.2. General Case of the RFVm Model

**Figure 3:**
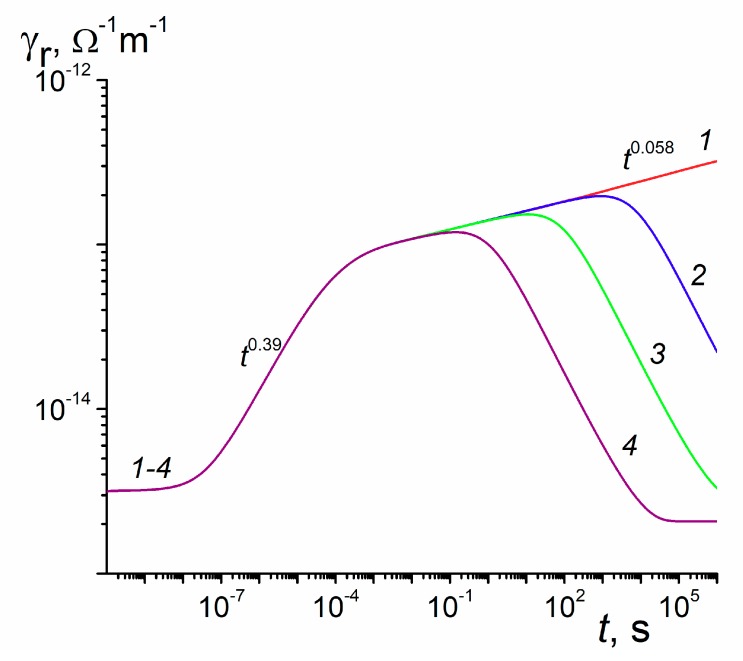
RIC curves calculated in a prototype polymer for the modified Rose-Fowler-Vaisberg (RFVm) model (semi-infinite geometry or alternatively, no transit time effects). Dose rate 10^14^ (1) 10^18^ (2) 10^20^ (3) and 10^22^ m^−3^·s^−1^ (4). Each curve was multiplied by a factor *ξ* = 10^20^ (m^−3^·s^−1^)/*g*_0_ (for curve (3), *ξ* is unity). Irradiation time 10^6^ s.

Now, we go beyond the *τ*_c_-approximation using specific parameters of the RFVm model: α_1_ = α = 0.5(*E*_1_ = 0.05 eV), α_2_ = 0.05 (*E*_2_ = 0.5 eV) and *E*_s_ = 0.28 eV. According to formula (8), we have *η* = 0.033. This value is rather close to *η* = *τ_0_*/*τ_c_* = 1/27 ≈ 0.037 for *τ*_c_ = 5.4 × 10^−10^ s used earlier. Numerical results are given in Figure 3. For curve (3), ${\overset{⌢}{\gamma }}_{p}=$3.2 × 10^−15^ Ω^−1^·m^−1^. Curve (1) shows that *β* as t→∞ is equal to 0.058 and is rather close to the expected value *β* = *α*_2_ = 0.05. At times around 20 μs *β* = 0.39 instead of its expected value *β* = *α*_1_ = 0.5. Processing curves (2–4) shows that the maximum conductivity *γ*_rm_ follows an algebraic dependence on the carrier generation rate $\gamma_{rm}\propto g_{0}^{\Delta }$ with Δ ≈ 0.87. According to formulas for dispersive transport (see References [9,14]), Δ = (1 + *α*_2_)^−1^ = 0.95 which differs appreciably from its numerical value 0.87. For reference, curve (3) has *γ*_rm_ = 1.5 × 10^−13^ Ω^−1^·m^−1^ (which is attained at 12.1 s). This value compares favorably with *j*_st_/*F*_0_ = 0.82 × 10^−13^ Ω^−1^·m^−1^ for curve (1) in Figure 2b (both quantities refer to the field 10^7^ V/m). This is expected as *η* values in both cases are rather close.

**Figure 4:**
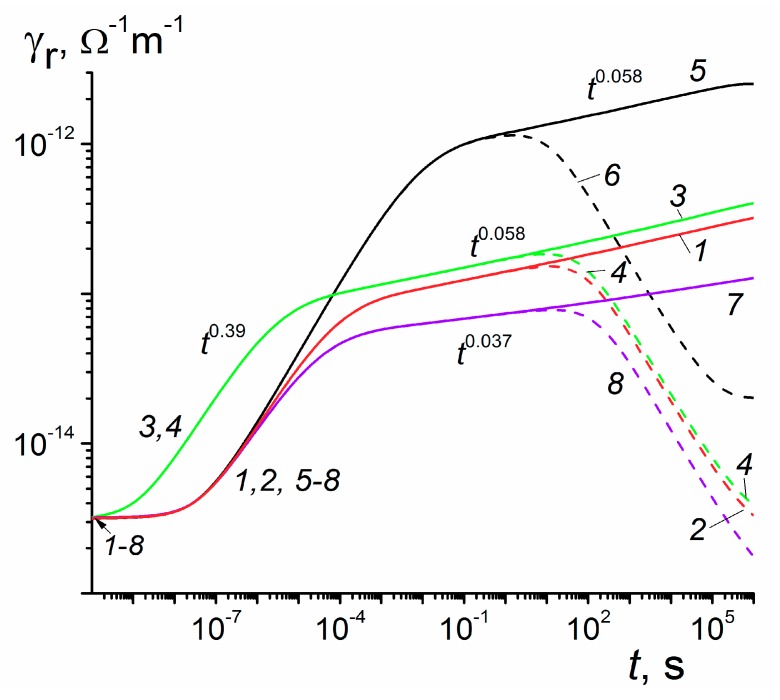
RIC curves calculated in a prototype polymer for the RFVm model (no transit time effects). Dose rate 10^14^ (1,3,5,7, solid) and 10^20^ m^−3^·s^−1^ (2,4,6,8, dash). Standard set of RFVm parameters (*1,2*); each parameter has been changed separately: *ν*_0_ = 9 × 10^8^ s^−1^ (3,4), *E*_s_ = 0.4 eV (5,6) and *α*_2_ = 0.02 (7,8). Curves for *g*_0_ = 10^14^ m^−3^·s^−1^ are scaled as in Figure 3. Irradiation time 10^6^ s. Curve (1) represents curve (3) in Figure 3.

Now, we examine the effect that each of the RFVm parameters exerts on RIC transients (Figure 4). In this respect, the separation energy *E*_s_ clearly stands out (compare curves (1) and (5) and this circumstance should be taken into consideration in parameter fitting procedure. Lowering *α*_2_ to 0.02 as in curve (7) reduces current density and *β* (0.037) which exceeds the expected value 0.02 by almost two times. One should remember that theoretical and calculated values of *β* at early times differ markedly (0.5 versus 0.39, respectively).

Usually, the role of the frequency factor is not to be underestimated. In our case, its influence is quite different at short and long times. Indeed, at 1 μs conductivity increases by 3.3 times whereas at 10 s it rises only 1.2 times for *ν*_0_ increasing by a factor of 30 (compare curves (1) and (3). The conventional effect is 30^0.39^ = 3.8 and 30^0.058^ = 1.2 (estimates agree rather well). We see that a big gain in conductivity at early times is effectively lost at long irradiation times.

Dashed curves calculated for *g*_0_ = 10^20^ m^−3^·s^−1^ (the corresponding dose rate is about 1.6 Gy/s which is close to a minimum dose rate provided by ELA-65) clearly delineate the limits of the small-signal irradiation (10 s for all curves except curve (6) when it approaches 0.3 s).

## 5. Discussion

To our knowledge, there are no present-day studies of the RIC in polymers that aim to investigate its main characteristics in detail, if only vaguely reminiscent of our approach. Most publications report RIC data retrieved from experimental results relating to phenomena such as the electron charging of polymer films in which RIC could not be directly measured. Model of choice is a two-trap quasi-band formalism [19,20,21] now extensively used by the ONERA French group of researchers [22,23]. Such data are intended to solve the urgent engineering problems but fail to contribute to an understanding of the detailed nature of the RIC phenomenon in polymers.

As noted in the Introduction, to deal with carrier transport in commercial polymers, we applied the radiation-induced TOF-2 variant which was initially developed to study charge carrier transport in photoconductive PVK and MDPs [24,25]. In this technique, we used a square (step-function) pulse which allows a time-resolved analysis of the build-up part of current transient in addition to the traditional processing of current decay after a pulse end. Exactly this approach combined with the small-signal RIC measurements on a broad time scale revealed limitations of the conventional MT theories to describe carrier transport in some commercial polymers [13].

There is no direct relationship between the RIC and electron energy. In our practice, the standard electron energy is 50 keV but in the past we used 2 and 10 MeV electron beams. The RIC is generated by the time derivative of the absorbed energy converted into generation rate of charge carriers (electrons and holes). The real problem is to find an average absorbed energy over the whole film thickness (which depends critically on an electron energy and a sample thickness) and to relate it to the RIC using an appropriate model. Second, it is important to work with single pulses of electrons as pulse cycling leads to inevitable RIC degradation as a result of radiation damage to a test sample. In our practice, we had to study specifically pulse cycling effect and did it by distancing individual pulses by some minutes enough to make measurements of the RIC degradation.

To avoid unnecessary damage effects in RIC and transit time measurements, we worked only with fresh polymer samples for each irradiation run (exceptions are possible for pulse irradiations with total accumulated dose not exceeding 10 Gy [9]. The prime aim of the RIC theory is to relate this time dependent energy profile with the local current density converging finally into the RIC current appearing in the external circuit.

## 6. Conclusions

The model of RIC in polymers developed in the present paper solves, for the first time, a problem of fundamental disagreement in the framework of the classical RFV theory between pulsed and continuous irradiations observed in some important commercial polymers such as PET, Kapton, polyethelenenaphthalene (PEN) while in some others including LDPE, PVK or MDPs this approach worked well (see Figure 2 and Figure 3) To achieve this, we introduced an aggregate two-exponential trap distribution accounting for deep traps with or without energy distribution allowing to describe unusual temporal behavior of the RIC under continuous irradiation. The physical origin of deep traps needs further clarification We developed a universal computer code and described how to fit experimental data by a tedious trial and error method. Our theoretical endeavor has become possible through almost 30 years of experimental RIC studies using an electron gun ELA-50 culminating in a remarkable paper [13] in which the above mentioned fundamental contradiction has been made crystal clear. We conclude that the RIC in polymers must be studied under pulsed and long-time small-signal irradiation in laboratory conditions as described in Reference [13] and should be interpreted in terms of the proposed RFVm (or RFV) models using appropriate computer codes to retrieve model parameters.

TOF effects predicted by the RFVm formalism are really extraordinary: they are almost nonexistent at weak fields and do appear only at very strong fields (see Figure 1) when their explanation in terms of a drift mobility [13,15] is totally misleading, in which case they should be interpreted as a complex field effect described by the model presented in this paper.

## References

[1] Pai D.M. (1970). Transient photoconductivity in poly(N-vinylcarbazole). J. Chem. Phys..

[2] Karl N., Kraft K.-H., Marktanner J., Münch M., Schatz F., Stehle R., Uhde H.-M. (1999). Fast electronic transport in organic molecular solids?. J. Vac. Sci. Technol. A.

[3] Scher H., Montroll E.W. (1975). Anomalous transit-time dispersion in amorphous solids. Phys. Rev. B.

[4] Arkhipov V.I., Iovu M.S., Rudenko A.I., Shutov S.D. (1979). An analysis of the dispersive charge transport in vitrous 0.55 As_2_Se_3_. Phys. Status Solidi A.

[5] Tiedje T., Rose A. (1981). A physical interpretation of dispersive transport in disordered semiconductors. Solid State Commun..

[6] Bässler H. (1993). Charge transport in disordered organic photoconductors. Phys. Stat. Sol. B.

[7] Borsenberger P.M., Weiss D.S. (1998). Organic Photoreceptors for Xerography.

[8] Novikov S.V., Dunlap D.H., Kenkre V.M., Parris P.E., Vannikov A.V. (1998). Essential role of correlations in governing charge transport in disorfered organic materials. Phys. Rev. Lett..

[9] Tyutnev A.P., Saenko V.S., Pozhidaev E.D., Ikhsanov R. (2015). Experimental and theoretical studies of radiation-induced conductivity in spacecraft polymers. IEEE Trans. Plasma Sci..

[10] Tyutnev A.P., Saenko V.S., Zhadov A.D., Pozhidaev E.D. (2019). Radiation-induced conductivity in Kapton-like polymers featuring conductivity rising with an accumulating dose. IEEE Trans. Plasma Sci..

[11] Tyutnev A.P., Saenko V.S., Zhadov A.D., Pozhidaev E.D. (2019). Time-resolved radiation-induced conductivity of polymers using the multiple trapping formalism. Polymers.

[12] Nikitenko V.R., Strikhanov M.N. (2014). Transport level in disordered organics:an analytic model and Monte-Carlo simulations. J. Appl. Phys..

[13] Tyutnev A.P., Saenko V.S., Ikhsanov R.S., Krouk E.A. (2019). Radiation-induced conductivity in polymers under pulsed and long-time small-signal irradiations combined to determine their step-function response. J. Appl. Phys..

[14] Arkhipov V.I. (1993). An adiabatic model of trap-controlled dispersive transport and recombination. J. Non-Cryst. Solids.

[15] Hughes R.C. Charge transport by photocarriers in polymeric films. Proceedings of the 2nd International Conference on Electrophotography.

[16] Kurtz S.R., Hughes R.C. (1983). Radiation-induced photoconductivity in polymers: Polyvinylfluoride compared with polyethylene terephthalate. J. Appl. Phys..

[17] Kurtz S.R., Arnold C., Hughes R.C. (1983). Effect of chemical doping on the radiation-induced conductivity of polyethylene terephthalate. Appl. Phys. Lett..

[18] Tyutnev A.P., Abramov V.N., Dubenskov P.I., Saenko V.S., Vannikov A.V., Pozhidaev E.D. (1986). Time-resolved nanosecond radiation-induced conductivity in polymers. Acta Polym..

[19] Gross B., Faria R.M., Ferreira G.F.L. (1981). Radiation-induced conductivity in Teflon irradiated by x-rays. J. Appl. Phys..

[20] Filho R.G., Gross B. (1989). Time-resolved x-ray conductivity in polyethyleneterephthalate. J. Appl. Phys..

[21] Sessler G.M., Figueiredo M.T., Ferreira G.F.L. (2004). Models of charge transport in electron-beam irradiated insulators. IEEE Trans. Diel. Electr. Insul..

[22] Molinie P., Dessante P., Hanna R., Paulmier T., Dirassen B., Belhaj M., Payan D., Balcon N. (2012). Polyimide and FEP charging behavior under multienergetic electron-beam irradiation. IEEE Trans. Dielectr. Electr. Insul..

[23] Paulmier T., Dirassen B., Payan D., Arnaout M. (2017). Analysis of charge transport and ionization effect in space used polymers under high energy electron irradiation. IEEE Trans. Plasma Sci..

[24] Dunlap D.H., Schein L.B., Tyutnev A.P., Saenko V.S., Pozhidaev E.D., Parris P.E., Weiss D.S. (2010). Two-layer multiple trapping model for universal current transients in molecilarly doped polymers. J. Phys. Chem. C.

[25] Tyutnev A.P., Weiss D.S., Dunlap D.H., Saenko V.S. (2014). Time-of-flight current shapes in mokecularly doped polymers:effects of sample thickness and irradiation side and carrier generation width. J. Phys. Chem. C.

